# New highland distribution records of multiple *Anopheles *species in the Ecuadorian Andes

**DOI:** 10.1186/1475-2875-10-236

**Published:** 2011-08-11

**Authors:** Lauren L Pinault, Fiona F Hunter

**Affiliations:** 1 Department of Biological Sciences, Brock University, F211 Mackenzie-Chown Complex, 500 Glenridge Avenue, St. Catharines, Canada, L2S 3A1

## Abstract

**Background:**

Several recent climate change reviews have stressed the possibility of some malaria vectors occupying regions of higher altitudes than previously recorded. Indeed, highland malaria has been observed in several African nations, possibly attributable to changes in land use, vector control and local climate. This study attempts to expand the current knowledge of the distribution of common *Anopheles *species in Ecuador, with particular attention to highland regions (> 500 m) of the Andes.

**Methods:**

Extensive field collections of larvae were undertaken in 2008, 2009 and 2010 throughout all regions of Ecuador (except the lower-altitude Amazonian plain) and compared to historical distribution maps reproduced from the 1940s. Larvae were identified using both a morphological key and sequencing of the 800 bp region of the CO1 mitochondrial gene. In addition, spatial statistics (Getis-Ord Hotspot Analysis: Gi*) were used to determine high and low-density clusters of each species in Ecuador.

**Results:**

Distributions have been updated for five species of *Anopheles *in Ecuador: *Anopheles albimanus*, *Anopheles pseudopunctipennis*, *Anopheles punctimacula*, *Anopheles eiseni *and *Anopheles oswaldoi s.l*.. Historical maps indicate that *An. pseudopunctipennis *used to be widespread in highland Andean valleys, while other species were completely restricted to lowland areas. By comparison, updated maps for the other four collected species show higher maximum elevations and/or more widespread distributions in highland regions than previously recorded. Gi* analysis determined some highland hot spots for *An. albimanus*, but only cold spots for all other species.

**Conclusions:**

This study documents the establishment of multiple anopheline species in high altitude regions of Ecuador, often in areas where malaria eradication programs are not focused.

## Background

Recently, there has been growing concern over the shifting distribution of malaria vectors due to land use alteration, changes to vector control measures, insecticide resistance, malaria treatment resistance, as well as local climate change [1-8]. Many reviews have discussed the hypothetical effects of climate change on insect-borne diseases, and some of these have stated the concern that malaria might be able to move into higher altitudes in the Andes, potentially affecting large cities such as Quito (2,800 m) [8-11]. Already, high-altitude malaria transmission has been recorded in a town in Bolivia at 2,300 m, vectored by *Anopheles pseudopunctipennis *[12]. However, the effects of climate change, such as rainfall, have only been weakly associated with cases of highland malaria [13,14], while land use alteration, changes to vector control programs and drug resistance have been more powerful drivers of highland malaria in African countries such as Kenya, Uganda, Tanzania, Madagascar, and Rwanda [1-7,15].

The resurgence of anopheline vectors in highland regions underlines the importance of studies that determine the distribution of *Anopheles *species in highland areas. The purpose of this paper is to identify the extent of previously lowland-restricted malaria vectors within highland regions of the Andes and to update distribution maps of the most common *Anopheles *species in Ecuador. These maps will identify highland areas, as well as low-altitude areas where *Anopheles *were recently collected, and thereby provide valuable data for malaria control in the country. Although other anopheline species have been reported in Ecuador [16,17], only five species were collected during the three years of intensive field work: *Anopheles albimanus*, *An. pseudopunctipennis*, *Anopheles punctimacula, Anopheles eiseni *and *Anopheles oswaldoi s.l*.. All five species are also found in other countries in Central and South America [18-20].

*Anopheles albimanus *has been considered a low-altitude (< 500 m) species that is believed to have become more abundant through the irrigation of low-lying areas [18]. Although there is one historical, anecdotal record of *An. albimanus *transmitting malaria at 1,800 m in southern Ecuador in the 1940s, the species has otherwise been considered an exclusively lowland species [21]. Currently, it is the primary malaria vector on the coast of Mexico and Belize [19,22-24]. *Anopheles albimanus *is distributed across the northern and Pacific coasts of Colombia [25-29] and Peru [30,31]. Historically (during the 1940s), *An. albimanus *was found as far north as Texas and Florida, USA [32] and throughout Central America, where it was collected in one locality at 1,000 m altitude [33-36]. It also was a main vector in Cuba and the West Indies [37], and was collected in South America along the northern Pacific coast, especially in the area surrounding Guayaquil, Ecuador [16,21]. *Anopheles albimanus *is not currently considered a species complex although there is some genetic variation among distinct populations from Pleistocene geographic fragmentation, even within Colombia (between the Caribbean and Pacific coasts) and Panama (between eastern and western populations) [26,29,38,39]. Further studies are required to determine if internal transcribed spacer 2 sequences from Central America provide evidence of rare cryptic species [39].

*Anopheles pseudopunctipennis *has historically been incriminated as the primary and often only malaria vector in highland Andean and Mexican valleys [21,34,40,41]. In the late 1940s, Levi-Castillo [41] documented the mosquito control efforts against *An. pseudopunctipennis *in highland regions near Quito, Ecuador. Since then, *An. pseudopunctipennis *has been documented as a coastal and also piedmont vector that is usually found up to 1,500 m in altitude [18]. Recently, *An. pseudopunctipennis *has been documented at extremely high altitudes (up to 2,800 m) in Bolivia [12,42]. Along with *An. albimanus*, *An. pseudopunctipennis *is one of the main vectors in the southern USA [43], in the foothills of Mexico [24], throughout Panama [44], and is also a main vector across Central America [22,43], western South America, Trinidad and Tobago, Haiti and other Caribbean islands [43]. When the population structure is examined across its distribution, *An. pseudopunctipennis *is a group of two sibling species with the greatest genetic differences being between the Central and South American populations [45,46].

*Anopheles punctimacula *is considered a possible secondary malaria vector along the coast of northern South America into Bolivia [18,30,44,47]. It was collected in the 1940s on the coast of Peru [48].

*Anopheles eiseni *was historically distributed across much of South and Central America, northward to southern Mexico [33-35,37], as well as in Pará, Brazil [49]. It is currently reported from Amazonian Brazil [20], Bolivia [47] and Central America [50].

*Anopheles oswaldoi *is an important malaria vector and species complex in the Peruvian, Colombian and Brazilian Amazon, where it often co-occurs with *Anopheles darlingi *[19,30,51-53], as well as occurring north into Panama [44]. Genetically, *An. oswaldoi s.l*. does not differ substantially among the northern parts of the Amazon, including Colombia, Venezuela and northern Brazil, although *An. oswaldoi *has been reported to be a species complex over the larger extent of its overall distribution [54]. Historically, *An. oswaldoi *has been collected in Panama, Trinidad and Costa Rica [37].

This paper outlines current distributions for the five most frequently collected species of *Anopheles *in Ecuador. For all species except *An. eiseni*, Getis-Ord Hotspot Analysis [55] was conducted to determine clusters of high and low-density larval populations. Additionally, to place the species distributions in an historical context (when highland malaria was last widespread in Ecuador), current distributions are compared to maps adapted from those compiled by Levi-Castillo [16] and Montalvan [21].

## Methods

**Figure 1:**
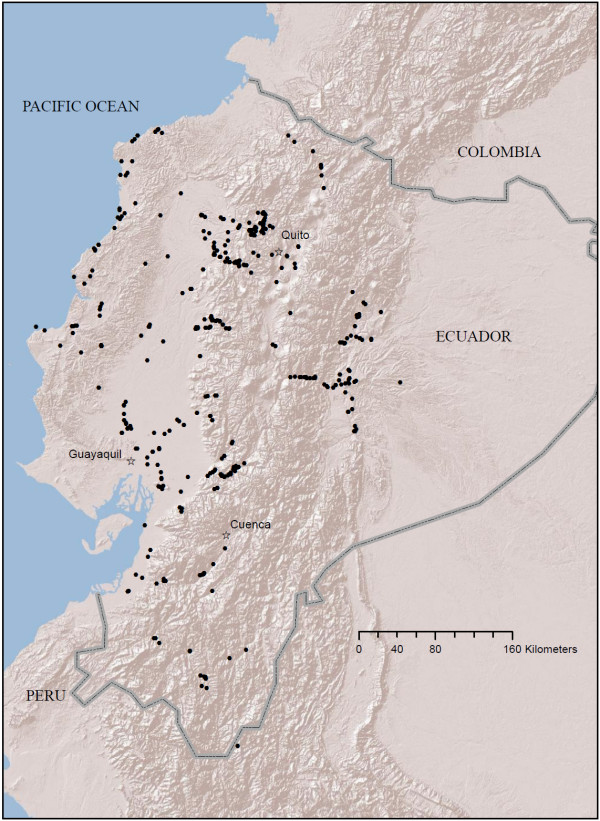
**Map of all attempted larval collection localities in Ecuador (black circles), between 2008 - 2010**. Map prepared in ArcGIS v.9.3 with ESRI World Terrain base [59].

Extensive field collections of *Anopheles *larvae were made in 2008, 2009 and 2010 throughout Ecuador (Figure 1). A strong effort was made to collect *Anopheles *larvae from sites at higher altitudes (i.e. > 500 m) than normally would be considered. Larvae were collected in 438 potential habitats that were encountered in all road-accessible regions of the country, except in the lowland Amazonian basin where road access was poor (Figure 1). Potential habitats were defined as any water source where the surface of the water was not moving, or moving very slowly (e.g., pond, river edge with algae). In each habitat, researchers sampled larvae using a 13 cm-diameter plastic dipping cup by skimming the surface of the water a standardized 30 times, extracting *Anopheles *larvae with a plastic pipette, and placing the larvae in 95% ethanol for transportation to the laboratory. In each habitat, latitude, longitude and altitude were recorded with a GPS unit (Garmin GPSmap76).

Large intact *Anopheles *larvae were identified using the morphology-based key of Gorham *et al *[17] supplemented with the description of *Anopheles trinkae *in Faran [56]. To confirm the morphological identifications of large, damaged larvae and small larvae too difficult to identify, a small (800 bp) region of the cytochrome oxidase 1 (CO1) gene was sequenced using the following procedure, and compared the sequences to those of easily identifiable large, intact larvae.

Larval bodies were ground with a small pestle in Lysis C solution and DNA was eluted into 30 μL elution buffer, using the GeneElute TM Mammalian Genomic DNA Miniprep Kit (Sigma-Aldrich). Primers for the CO1 800 bp region were C1-J-2195 (F) and UEA (R) [57,58]. The PCR reaction mixture was composed of: 2.3 μL buffer, 1.3 μL × 50 mM MgSO_4_, 0.25 μL × 10 μmol F and R primers, 0.3 μL DNTPs, and 0.3 μL Taq Polymerase, and was run with 5.0 μL DNA extraction mixture. The PCR ran for 30 cycles for 2.5 minutes at 95°C, 45 seconds at 51°C, 1 minute at 72°C, and a final cycle of 10 minutes at 72°C. Degraded DNA was run for 40 cycles of the same regime.

PCR products were sent for sequencing at Genome Quebec, McGill University and aligned after trimming in ClustalW (European Bioinformatics Research Institute 2010). Consensus sequences for each species were determined from five larvae that were easily identified morphologically and provided high-quality sequences for comparison with unidentifiable larvae. Sequence consensuses were 97-100% within species and 84-90% among species, except for very low-quality sequences. In these cases, a positive match for the same species was approximately 10% greater than among species. Consensus sequences have been submitted to GenBank (accession numbers JN412826-JN412843). All specimens not destroyed for molecular work were deposited in the Ecuadorian National Insect Collection at the Pontificía Universidad Católica del Ecuador (PUCE) in 2009 and 2010.

All distribution maps were made using ArcGIS v9.3 software (ESRI 2009). The background layer used for all maps is the ESRI World Terrain Base [59]. For the current collection data, density (number of larvae per 30 dips) is indicated on the maps by different sizes of map points. For each species' current distribution, Getis-Ord Hot Spot Analysis (Gi*) [55] was conducted using larval density as the weighting factor. Significant hot spots and cold spots (high and low-density clusters of larval populations) are indicated in each distribution map. Historical collection data for four of the species were adapted from hand-drawn maps by Levi-Castillo [16] and Montalvan [21], by using the original town square in the city names when provided, or by using rivers and landmarks in the original maps to determine approximate coordinates in GoogleEarth (Google 2008).

## Results

**Figure 2:**
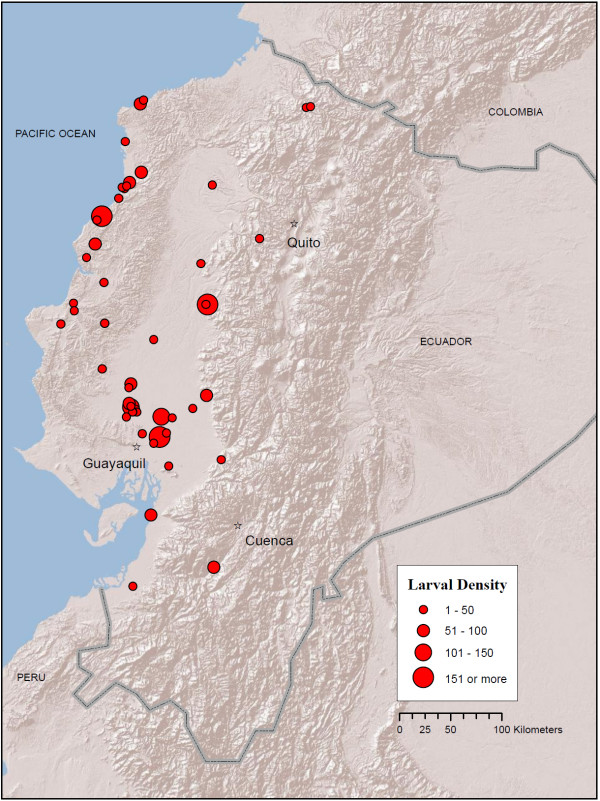
**Map of *An. albimanus *localities in Ecuador (red circles) from larval collections conducted between 2008 - 2010**. Circle size (legend) indicates mean number of larvae per dip in a 30-dip sample. Map prepared in ArcGIS v.9.3 with ESRI World Terrain base [59].

**Figure 3:**
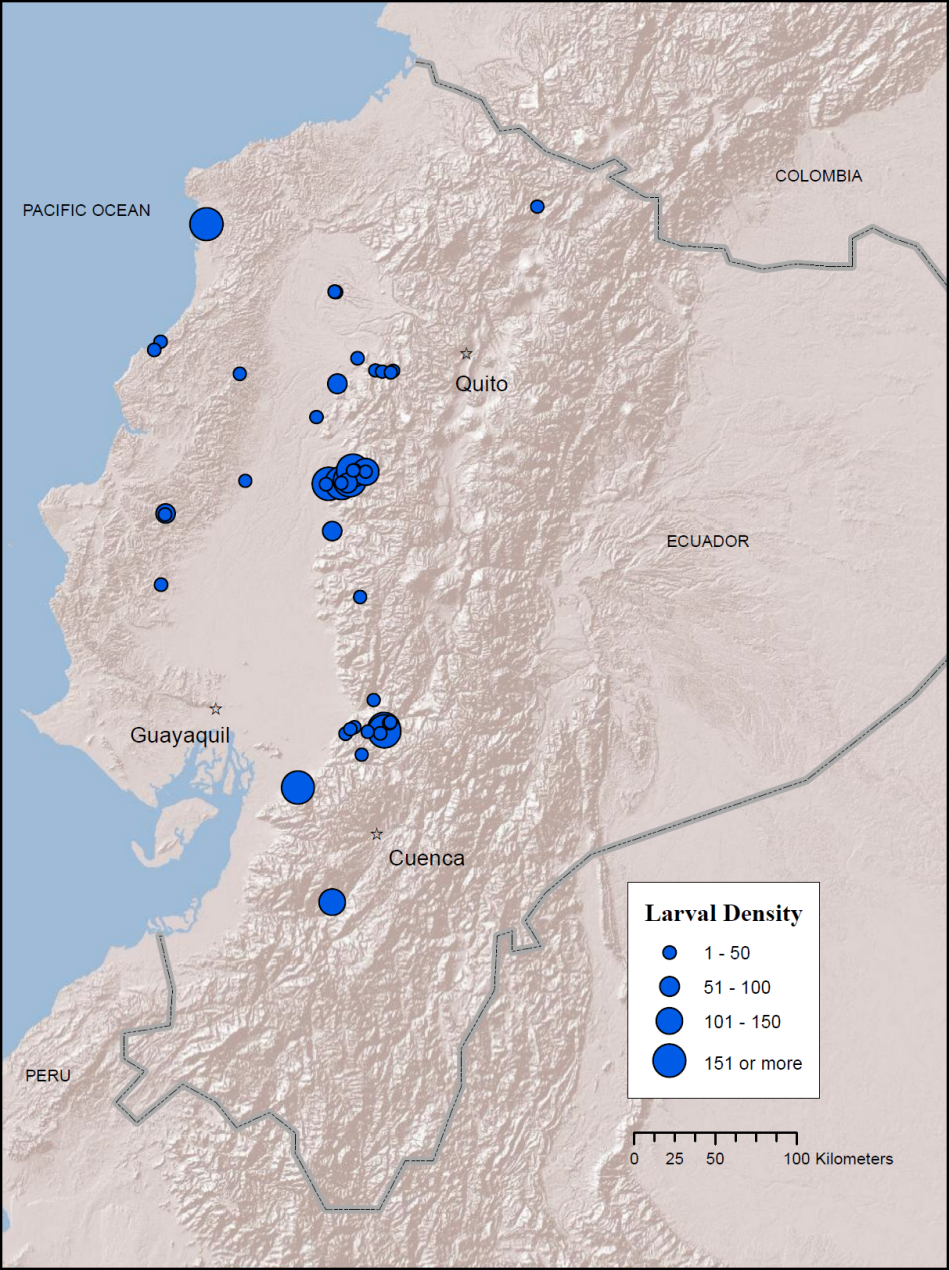
**Map of *An. pseudopunctipennis *localities in Ecuador (blue circles) from larval collections conducted between 2008 - 2010**. Circle size (legend) indicates mean number of larvae per dip in a 30-dip sample. Map prepared in ArcGIS v.9.3 with ESRI World Terrain base [59].

**Figure 4:**
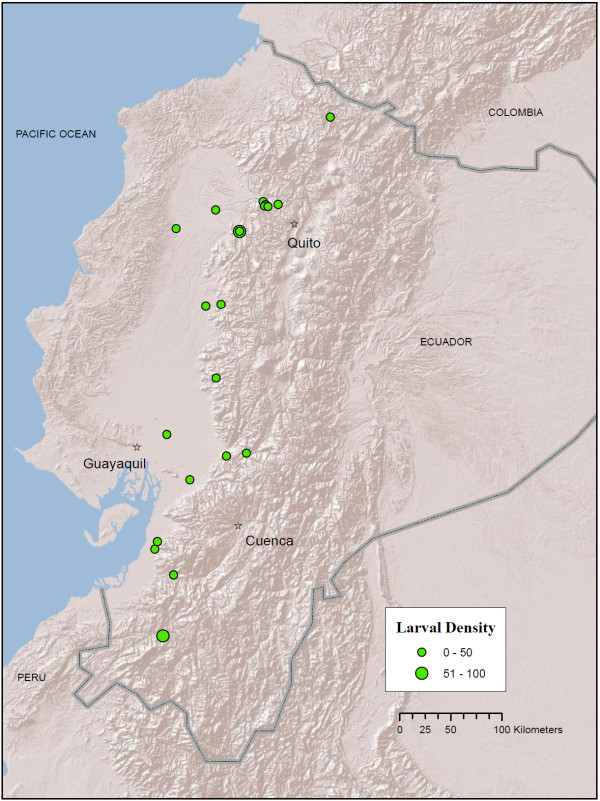
**Map of *An. punctimacula *localities in Ecuador (green circles) from larval collections conducted between 2008 - 2010**. Circle size (legend) indicates mean number of larvae per dip in a 30-dip sample. Map prepared in ArcGIS v.9.3 with ESRI World Terrain base [59].

**Figure 5:**
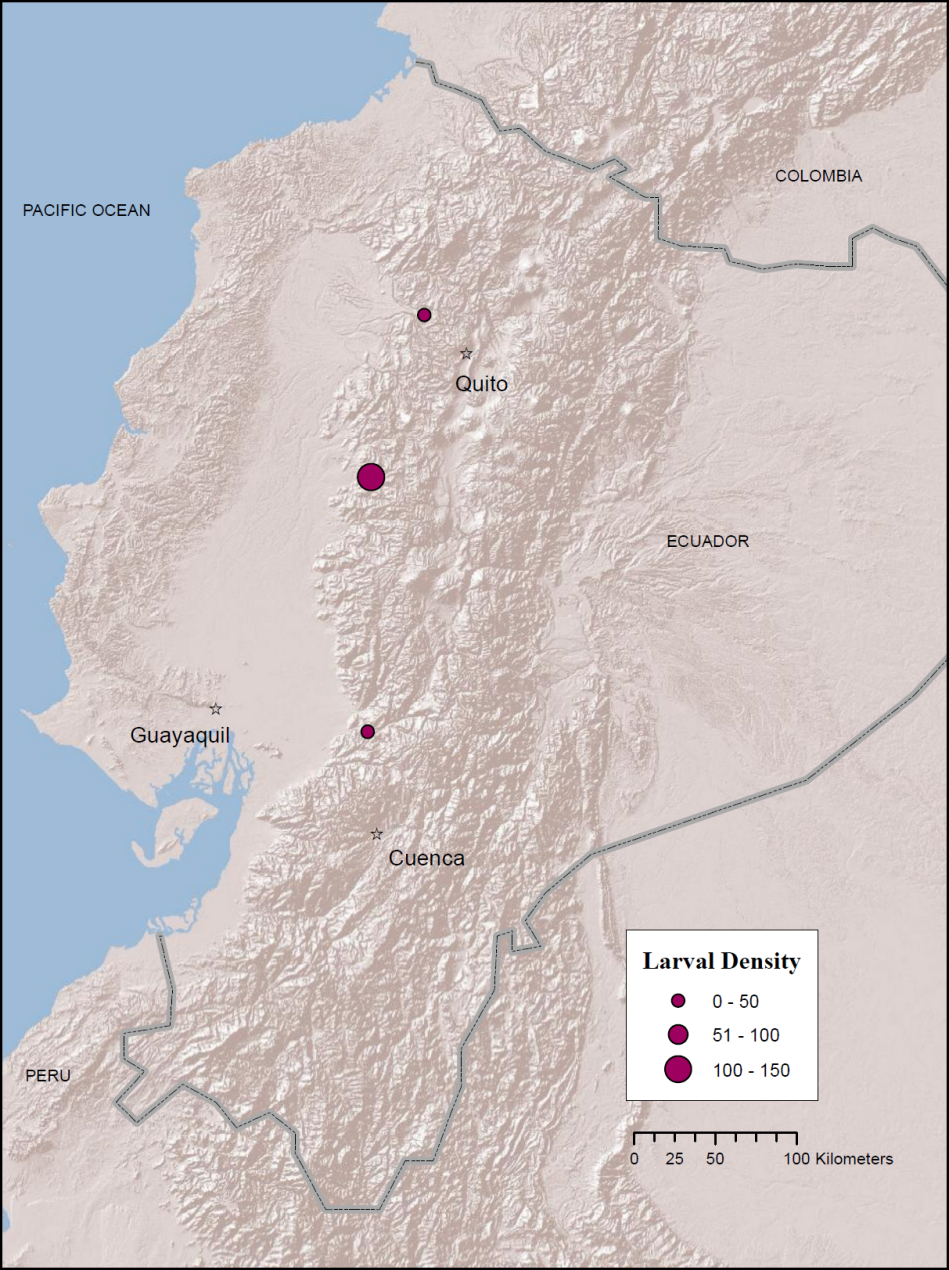
**Map of *An. eiseni *localities in Ecuador (maroon circles) from larval collections conducted between 2008 - 2010**. Circle size (legend) indicates mean number of larvae per dip in a 30-dip sample. Map prepared in ArcGIS v.9.3 with ESRI World Terrain base [59].

**Figure 6:**
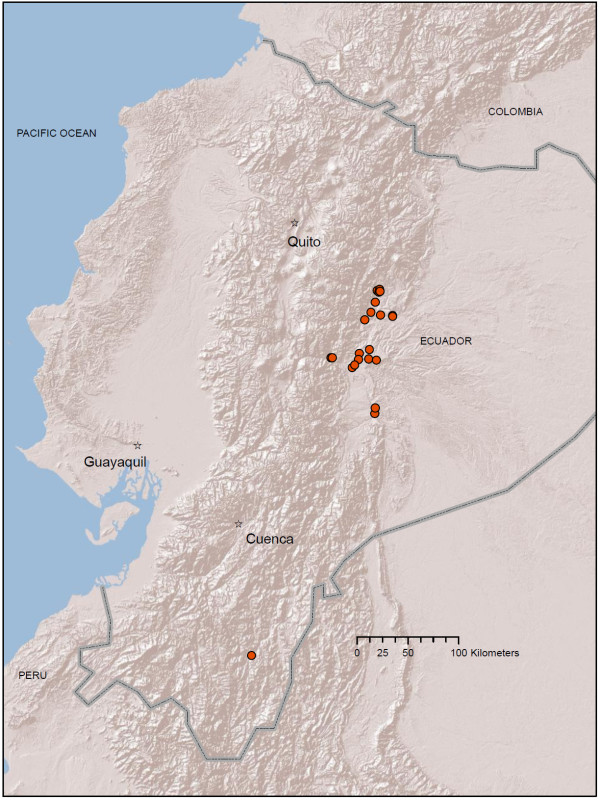
**Map of *An. oswaldoi s.l*. localities in Ecuador (orange circles) from larval collections conducted between 2008 - 2010**. All sample sizes are within 1-50 larvae per 30-dip sample. Map prepared in ArcGIS v.9.3 with ESRI World Terrain base [59].

**Figure 7:**
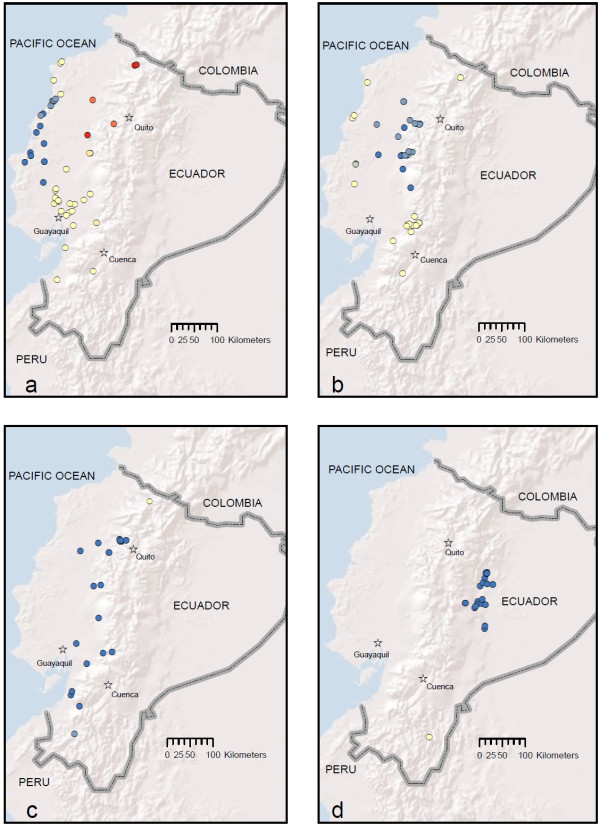
**Hot Spot Analysis (Getis-Ord) results for a) *An. albimanus*, b) *An. pseudopunctipennis*, c) *An. punctimacula *and d) *An. oswaldoi s.l***.. Red and dark orange circles indicate significant clusters of high larval density sites, whereas blue circles indicate significant clusters of low larval density sites (p < 0.05). Map prepared in ArcGIS v.9.3 with ESRI World Terrain base [59].

**Figure 8:**
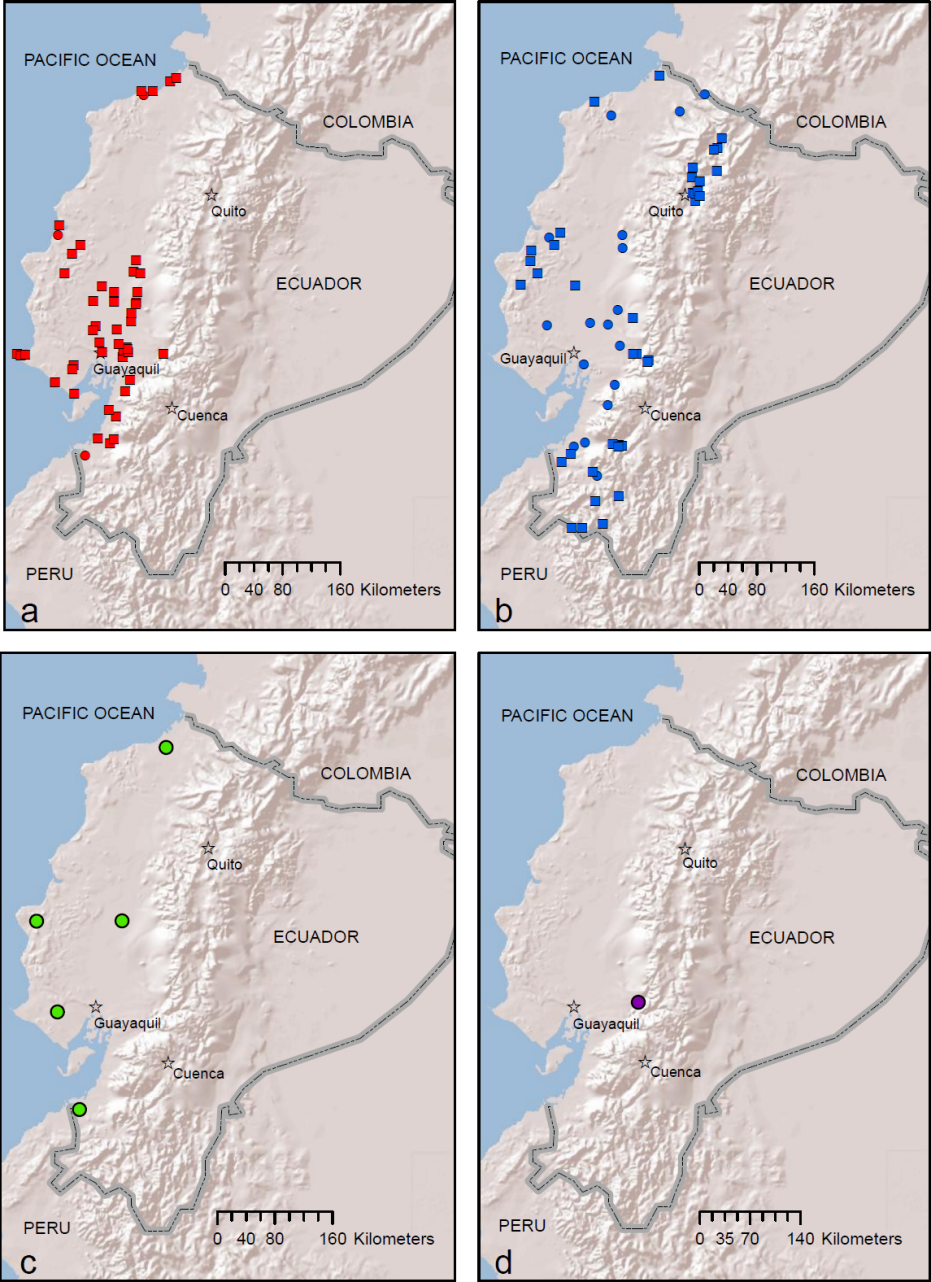
**Historical distribution during the 1940s of a) *An. albimanus*, b) *An. pseudopunctipennis*, c) *An. punctimacula *and d) *An. eiseni***. Data reproduced from historical maps by Levi-Castillo (circles) [16] and Montalvan (squares) [21]. Map prepared in ArcGIS v.9.3 with ESRI World Terrain base [59].

Current distribution maps for all five species are presented in Figures 2, 3, 4, 5 and 6. *Anopheles albimanus *larvae were collected in three highland localities in Ecuador: in the northern Mira River valley, Imbabura Province (767 and 832 m), near La Hesperia Biological Station, Pichincha Province (1366 m), and in the south of the country, near Girón, Azuay Province (1,541 m) (Figure 2; Additional file 1 Table S1). *Anopheles albimanus *was collected predominantly in the coastal region of Ecuador, most notably along the northern coast proper, and in the vast rice-growing region northeast of Guayaquil (Figure 2). At all latitudes, *An. albimanus *was also found in foothill regions, up to an altitude of 283 m (Figure 2). Gi* Hot Spot Analysis identified five significant hot spots for *An. albimanus *larvae (i.e., sites among clusters of other high-density sites): two in the Mira valley, Imbabura Province (Gi* = 4.37; p < 0.001), one in Puerto Quito, Pichincha Province (Gi* = 2.16; p = 0.03), one high-altitude site near La Hesperia Biological Station, Pichincha Province (Gi* = 2.56; p = 0.01) and one near Santo Domingo, in Santo Domingo de los Tsáchiles Province (Gi* = 3.056; p = 0.002) (Figure 7a). The analysis also identified 15 cold spots (i.e., sites among clusters of other low-density sites), all located along the north-western coast (Figure 7a). Clusters of hot and cold spots contained larvae from multiple instars and, therefore, do not represent sister larvae from the same females. In the 1940s, *An. albimanus *was ubiquitous on the coast of Ecuador, particularly in many areas surrounding Guayaquil (Figure 8a). However, it is important to note that in historical entomological collections, *An. albimanus *was not recorded at elevations higher than approximately 500 m (Figure 8a).

*Anopheles pseudopunctipennis *larvae were most common along the highway linking El Triunfo, Guayas Province, to Alausí, Chimborazo Province, up to a maximum altitude of 1,558 m, as well as the road linking Quevedo, Los Rios Province, to Pilaló, Cotopaxi Province, to a maximum altitude of 858 m (Figure 3; Additional file 1 Table S1). Other high-altitude localities include the Mira River valley, Imbabura Province (1,273 m), San Antonio, Bolívar Province (1,321 m), Unión del Toachi, Pichincha Province (835 m), Santa Isabel, Azuay Province (1,353 m) and Chilchil, Cañar Province (1,930 m) (Figure 3, Additional file 1 Table S1). *Anopheles pseudopunctipennis *larvae were collected in a few localities on the coastal plain of Ecuador, but the vast majority were collected in the lower parts of the Andes on the coastal side (Figure 3). Hot spot analysis did not identify any hot spots, but 23 significant cold spots (low-density clusters) were identified in the northern coastal region, with groups of these in the regions of Alluriquin, Pichincha Province, and La Maná, Cotopaxi Province (Figure 7b). Historical maps show that *An. pseudopunctipennis *was widespread in almost all coastal regions, as well as in highland areas, particularly in the south (Loja Province) and in the northern valleys surrounding Quito, Pichincha Province, and Ibarra, Imbabura Province (Figure 8b).

*Anopheles punctimacula *larvae were collected predominantly in the coastal-side foothills of the Andes within an approximate elevation range of 147-1,300 m, although they were also collected in a few localities on the coast (Figure 4). The species was often collected in areas near Mindo, Pichincha Province (1,105-1,312 m), but was also collected in Mira valley, Imbabura Province (1,234 m) and in a high-altitude site near Sibambe, Chimborazo Province (1,906 m) (Figure 4; Additional file 1 Table S1). No hot spots were identified in the Gi* analysis, although 19 cold spots were identified spread across most of *An. punctimacula*'s Ecuadorian distribution (Figure 7c). Interestingly, historical distributions of *An. punctimacula *place the species in four localities: three on the coast proper, and one on the coastal plain (Figure 8c).

*Anopheles eiseni *was collected at three high-altitude sites (1,206-1,873 m) on the coastal side of the Andes (Figure 5; Additional file 1 Table S1). Gi* Analysis was not possible due to the small number of localities. Interestingly, historical distributions place *An. eiseni *in one locality only (Figure 8d), very close to the current collection site near Tingo, Cotopaxi Province (Figure 5).

*Anopheles oswaldoi s.l*. was the only species collected on the Amazonian side of the mountains at altitudes greater than 416 m (Figure 6; Additional file 1 Table S1). The lower-altitude Amazonian plain was excluded from our study since these areas were not road-accessible and were also beyond the scope of this study (i.e., highland focus). Two higher-altitude localities were recorded for this species: Mera, Pastaza Province (1,233 m), and Río Verde, Tungurahua Province (1,230 m) (Figure 6; Additional file 1 Table S1). The species was abundant in areas surrounding Tena and Archidona, Napo Province, and Puyo, Pastaza Province, and was collected once in Zamora, Zamora-Chinchipe Province (Figure 6; Additional file 1 Table S1). Gi* analysis did not identify any hot spots for *An. oswaldoi s.l*., although 20 cold spots were identified in areas surrounding Tena, Napo Province and Puyo, Pastaza Province (Figure 7d). There are no historical records available for *An. oswaldoi s.l*.

## Discussion

Historically, *An. pseudopunctipennis *was abundant and widespread in highland areas, while *An. albimanus *and *An. punctimacula *were considered species generally restricted to lowland areas (< 500 m) on the coast [16,21]. It is clear from the maximum altitudes recorded for each of these species, as well as for *An. oswaldoi s.l*. and *An. eiseni*, that all five of the most common species of *Anopheles *are now residing in highland regions of the Ecuadorian Andes. Although no species on the coastal side was collected at altitudes exceeding 2,000 m, only a small change in environmental conditions at higher altitudes may be required for species to move into highland valleys (2,100-2,400 m) if suitable habitat is present. *Anopheles pseudopunctipennis *is physiologically able to survive at these altitudes, since it existed in highland valleys during the 1940s [16,21] and recently has been reported at these altitudes in Bolivia [12,42]. From the 1940s maps of *An. pseudopunctipennis *[16,21], and from recent reports in Bolivia [12,42], it must be at least physiologically possible for this species. Interestingly, no *An. pseudopunctipennis *larvae were observed in extremely high altitudes, as in Bolivia (2,000 - 2,800 m) [42], likely due to a combination of different land use, topography, and microclimate in highland Ecuador.

Lacking proper data sets, it is difficult to know whether *An. pseudopunctipennis *have occupied highland regions continuously since the 1940s. Levi-Castillo [41] documents the successful elimination of *An. pseudopunctipennis *from highland valleys near Quito through the use of powerful chemical insecticides and habitat elimination, and anecdotal evidence suggests that the species has not been widely present since then. However, it is possible that very small, undetected populations of this species have remained in highland regions since that time, as is believed to be the case for *Anopheles gambiae *in Tanzania [60].

As opposed to the study by Balls *et al *[61] in Tanzania, where steep topography limited available *An. gambiae *and *Anopheles funestus *larval habitat in highland regions [61,62], many suitable habitats are available in the Ecuadorian highlands: alongside roads, rivers and irrigation ditches. Although the amount of habitat available to mosquitoes is probably much less in steep areas, the habitat in highland regions is obviously sufficient to allow species to become established.

Hot spots and cold spots both indicate clusters of larval populations, and both are therefore important to identify foci of potential malaria transmission. Particularly troubling are the hot spots for *An. albimanus *in the region between Puerto Quito, Pichincha Province, and Santo Domingo, Santo Domingo de los Tsáchiles Province, where cases of malaria caused by both *Plasmodium vivax *and *Plasmodium falciparum *still occur [unpublished data, Ministerio de Salud Pública, Government of Ecuador]. Most low and moderate-elevation regions of Ecuador are affected by clusters of at least one species of *Anopheles*, which indicates that potential malaria vectors have already become established in many regions that have been previously considered completely malaria-free. As well, favourable climatic conditions one year could easily cause low larval densities (cold spots) to increase in population size and become hot spots, potentially leading to malaria outbreaks.

The establishment of various *Anopheles *mosquito species in highland regions highlights the importance of awareness by decision-makers and the general population. Those involved in malaria control in Ecuador will need to begin to consider higher-altitude regions (i.e., 1,000-2,000 m) as potential regions of malaria transmission by multiple vectors and to monitor suspicious illness accordingly. Further studies are necessary to determine the exact nature of the highland *Anopheles *larval habitat and the extent to which these species might further establish themselves in even higher-altitude regions in the future.

## Conclusions

Although *An. pseudopunctipennis *has been historically incriminated as the only widespread malaria vector in highland regions of the Ecuadorian Andes, present collections indicate that the distributions of *An. albimanus*, *An. punctimacula *and *An. oswaldoi s.l*. are encroaching into higher altitude regions, in some cases reaching higher maximum altitudes (1,541 m, 1,906 m, and 1,230 m, respectively). For all species, larvae were collected in highland regions in multiple localities within Ecuador. The establishment of multiple malaria vectors in the Ecuadorian highlands will add a greater degree of complexity to the prevention and/or eradication of malaria in highland regions.

## Competing interests

The authors declare that they have no competing interests.

## Authors' contributions

LLP contributed to the conception, study design, data collection in the field, entomological identification, data analysis, GIS mapping, and writing of the manuscript. FFH contributed to the conception, study design, entomological identification and revision of the manuscript. Both authors read and approved the final manuscript.

## Supplementary Material

Additional File 1 **Table S1 - Highland collection localities for *Anopheles *larvae in Ecuador during 2008, 2009 and 2010**. Altitudes and habitat types of *An. albimanus *(ALB), *An. pseudopunctipennis *(PSE), *An. punctimacula *(PUN), *An. eiseni *(EIS), and *An. oswaldoi s.l*. (OSW), larvae collected in highland (steep topography, > 500 m) regions of Ecuador are provided. Click here for file
