# Supplementary material for: New highland distribution records of multiple Anopheles species in the Ecuadorian Andes
Source: Malar J. 2011 Aug 11;10:236. doi: 10.1186/1475-2875-10-236 (PMC3176254; doi:10.1186/1475-2875-10-236)
Supplement: Additional File 1 — Table S1 - Highland collection localities for Anopheles larvae in Ecuador during 2008, 2009 and 2010. Altitudes and habitat types of An. albimanus (ALB), An. pseudopunctipennis (PSE), An. punctimacula (PUN), An. eiseni (EIS), and An. oswaldoi s.l. (OSW), larvae collected in highland (steep topography, > 500 m) regions of Ecuador are provided. [file 1475-2875-10-236-S1.DOCX]

## Additional File 1: Table S1 - Highland collection localities for *Anopheles* larvae in Ecuador during 2008, 2009 and 2010.

Altitudes and habitat types of *An. albimanus* (ALB), *An. pseudopunctipennis* (PSE), *An. punctimacula* (PUN), *An. eiseni* (EIS), and *An. oswaldoi* *s.l*. (OSW), larvae collected in highland (steep topography, >500m) regions of Ecuador are provided.

| Province | Spp. | Locality | Latitude | Longitude | Alt. (m) | | | Habitat |
| --- | --- | --- | --- | --- | --- | --- | --- | --- |
| Azuay | ALB | Near Girón | S03°15.06ʹ | W79°14.23ʹ | 1541 | Pond | | |
|  | PSE | Near Santa Isabel | S03°15.67ʹ | W79°16.41ʹ | 1353 | River eddy | | |
|  | PSE | Near El Inca | S02°37.54ʹ | W79°27.74ʹ | 805 | Pool | | |
|  | PSE | San Antonio | S01°34.28ʹ | W79°07.76ʹ | 1321 | WFD^1^ | | |
|  | PSE | Near El Triunfo | S02°15.89ʹ | W79°57.06ʹ | 592 | Pond | | |
|  | PSE | Chilchil | S02°26.68ʹ | W79°06.54ʹ | 1930 | River edge | | |
| Bolívar | PUN | Caluma | S01°34.88ʹ | W79°13.18ʹ | 517 | Pond | | |
| Chimborazo | PSE | Near Huigra | S02°19.00ʹ | W79°04.55ʹ | 1206 | River eddy | | |
|  | PSE | Near Huigra | S02°18.84ʹ | W78°59.06ʹ | 1389 | WFD^1^ | | |
|  | PSE | Near Huigra | S02°19.48ʹ | W79°00.49ʹ | 1426 | Rock pool | | |
|  | PSE | SW of Huigra | S02°18.89ʹ | W78°59.87ʹ | 1387 | WFD^1^ | | |
|  | PSE | NE of Huigra | S02°16.09ʹ | W78°57.46ʹ | 1558 | Road ditch | | |
|  | PSE | NE of Huigra | S02°16.07ʹ | W78°57.43ʹ | 1553 | Road ditch | | |
|  | PSE | Near Suropata | S02°08.55ʹ | W79°02.59ʹ | 674 | River | | |
|  | PSE | West of Tingo | S00°52.60ʹ | W79°05.20ʹ | 858 | WFD^1^ | | |
|  | PSE | Near La Maná | S00°52.61ʹ | W79°05.19ʹ | 851 | River WFD^1^ | | |
|  | PSE | Near La Maná | S00°52.27ʹ | W79°09.52ʹ | 751 | River WFD^1^ | | |
|  | PUN | Near Sibambe | S02°14.65ʹ | W78°57.22ʹ | 1906 | River eddy | | |
|  | EIS | Near Huigra | S02°19.04ʹ | W79°04.55ʹ | 1206 | Road ditch | | |
| Cotopaxi | EIS | Near Tingo | S00°54.41ʹ | W79°03.51ʹ | 1423 | Cement pool | | |
| El Oro | PUN | Near Uzhcurrum | S03°18.98ʹ | W79°35.69ʹ | 539 | Road ditch | | |
| Guayas | PUN | El Triunfo | S02°16.08ʹ | W79°07.70ʹ | 531 | River eddy | | |
| Imbabura | ALB | Santa Rita | N00°48.71ʹ | W78°23.36ʹ | 767 | Tire tracks | | |
|  | ALB | Near Lita | N00°48.60ʹ | W78°19.39ʹ | 832 | Ditch | | |
|  | PSE | Mira River valley | N00°35.55ʹ | W78°08.25ʹ | 1273 | Road ditch | | |
|  | PUN | Lita-Salinas hwy. | N00°43.32ʹ | W78°13.92ʹ | 1234 | WFD^1^ | | |
| Loja | PUN | West of Chaguarpamba | S03°51.33ʹ | W79°41.44ʹ | 680 | River pool | | |
| Napo | OSW | Near Archidona | S00°55.45ʹ | W77°44.32ʹ | 560 | Pond | | |
|  | OSW | Mondayacu | S00°48.47ʹ | W77°46.64ʹ | 907 | WFD^1^ | | |
|  | OSW | Mondayacu | S00°49.62ʹ | W77°46.58ʹ | 837 | Road ditch | | |
|  | OSW | Cotundo valley | S00°50.00ʹ | W77°47.05ʹ | 779 | WFD^1^ | | |
|  | OSW | Near Misahuallí | S00°49.21ʹ | W77°48.02ʹ | 835 | Pond | | |
|  | OSW | Road to Misahualli | S01°02.15ʹ | W77°46.21ʹ | 513 | WFD^1^ | | |
|  | OSW | Bajo Talug (Cando) | S01°04.72ʹ | W77°54.63ʹ | 567 | Road ditch | | |
|  | OSW | Pano | S01°00.77ʹ | W77°51.27ʹ | 566 | Road ditch | | |
|  |  |  |  |  |  |  | | |
|  |  |  |  |  |  | |  | |
|  |  |  |  |  |  | |  | |
|  |  |  |  |  |  | |  | |
| Province | Spp. | Locality | Latitude | Longitude | Alt. (m) | | Habitat | |
|  |  |  |  |  |  | |  | |
|  | OSW | Near Puyo | S01°25.82ʹ | W77°57.88ʹ | 998 | Marsh | | |
|  | OSW | Puyo | S01°28.63ʹ | W77°59.88ʹ | 938 | Pond | | |
|  | OSW | Near Puyo | S01°30.06ʹ | W78°01.20ʹ | 938 | Ditch | | |
|  | OSW | Near Mera | S01°24.83ʹ | W78°12.45ʹ | 1223 | Road ditch | | |
|  | OSW | Sharupi centre | S01°54.55ʹ | W77°49.38ʹ | 665 | Pond | | |
|  | OSW | Kunkuki | S01°51.55ʹ | W77°48.99ʹ | 894 | Pond | | |
|  | OSW | San Ramon | S01°25.43ʹ | W77°52.46ʹ | 991 | River eddy | | |
|  | OSW | Simon Bolivar | S01°26.21ʹ | W77°48.43ʹ | 1045 | Pond | | |
| Pichincha | ALB | San Vicente | S01°20.49ʹ | W77°52.03ʹ | 1006 | River eddy | | |
|  | PSE | South of U. Toachi | S00°19.66ʹ | W78°56.91ʹ | 835 | River eddy | | |
|  | PSE | Near U.Toachi | S00°19.57ʹ | W78°56.91ʹ | 805 | Pool | | |
|  | PUN | Mindo | S00°04.12ʹ | W78°45.74ʹ | 1289 | Pond | | |
|  | PUN | Mindo | S00°02.94ʹ | W78°40.44ʹ | 1312 | Pond | | |
|  | PUN | Mindo valley | S00°01.48ʹ | W78°48.40ʹ | 1105 | River pool | | |
|  | PUN | Road Mindo–Lloa | S00°03.88ʹ | W78°47.60ʹ | 1280 | Road ditch | | |
|  | PUN | North Alluriquin | S00°14.92ʹ | W79°00.92ʹ | 847 | River eddy | | |
|  | PUN | Near Mindo | S00°03.53ʹ | W78°46.77ʹ | 1261 | Road ditch | | |
|  | PUN | Near Mindo | S00°03.09ʹ | W78°47.09ʹ | 1239 | Road ditch | | |
|  | EIS | Mindo fish farm | S00°04.15ʹ | W78°45.71ʹ | 1873 | Fish pond | | |
| S. Domingo d.l Tsachiles | PSE | City of Santo Domingo | S00°14.92ʹ | W79°08.06ʹ | 532 | Marsh | | |
|  | PSE | Alluriquin | S00°19.41ʹ | W78°59.86ʹ | 740 | River eddy | | |
|  | PSE | Leila | S00°18.96ʹ | W79°02.00ʹ | 778 | Pool | | |
|  | PUN | La Florida | S00°17.20ʹ | W79°00.85ʹ | 847 | River pool | | |
| Tungurahua | OSW | Río Negro | S01°24.84ʹ | W78°11.81ʹ | 1230 | Road ditch | | |
| Zamora-Chinchipe | OSW | Near Zamora | S04°02.85ʹ | W78°54.69ʹ | 893 | Flooded area | | |

^1^WFD = Water-filled depression
